# Motion-State-Aware Adaptive Step-Length Smartphone PDR for GPS-Denied Pedestrian Localization

**DOI:** 10.3390/s26154915

**Published:** 2026-08-04

**Authors:** Huabang Liu, Wanfeng Dou, Hexing Wang

**Affiliations:** 1 School of Mathematics and Statistics, Northeastern University at Qinhuangdao, Qinhuangdao 066000, China; 2 School of Computer and Electronic Information, Nanjing Normal University, Nanjing 210023, China; 3 Jiangsu Intelligent Information Technology and Software Engineering Laboratory, Nanjing Normal University, Nanjing 210023, China; 4 School of Computer and Communication Engineering, Northeastern University at Qinhuangdao, Qinhuangdao 066000, China

**Keywords:** pedestrian dead reckoning (PDR), joint motion state, decoder-only Transformer, adaptive step-length modeling, smartphone sensors, GPS-denied pedestrian localization

## Abstract

Smartphone-based pedestrian dead reckoning (PDR) provides an infrastructure-free solution for two-dimensional (2D) planar localization in GPS-denied environments, but its open-loop nature makes it sensitive to accumulated step-length and heading errors. These errors grow when pedestrian actions and phone carrying modes change, because conventional methods use a fixed step-length model with a constant Weinberg coefficient. This paper proposes a motion-state-aware PDR method with two key designs. First, a joint motion state defined by action type and carrying mode is recognized from smartphone sensor data using a random-forest classifier. Second, the Weinberg coefficient is modeled through two adaptive variants: a state-wise linear model as the main lightweight adaptation mechanism, and a Transformer-enhanced extension that uses historical step-feature sequences to provide additional temporal smoothing for the per-step coefficient *K*. Both variants keep the predicted coefficient inside the Weinberg equation to preserve the physical structure of step-length estimation, with offline training minimizing the distance error over each calibrated segment. Heading is estimated by fusing gyroscope increments and magnetometer observations to improve continuity under magnetic disturbance. Experiments on routes with frequent motion-state transitions, including a representative indoor corridor with magnetic disturbance and turns, compare a fixed-parameter baseline and two established adaptive step-length baselines against the proposed variants using coefficient-modeling diagnostics and trajectory-level metrics. More challenging deployments such as underground or multi-floor environments are left for future work.

## 1. Introduction

Smartphone-based pedestrian dead reckoning (PDR) offers a self-contained and infrastructure-free solution for pedestrian localization in GPS-denied environments [1,2,3]. Typical target settings include large public buildings (e.g., malls, airports, hospitals), underground facilities (e.g., metro stations, tunnels, parking structures), and other GNSS-degraded areas where installing dedicated infrastructure or collecting radio fingerprints is costly or impractical. In such settings, PDR can operate using only the inertial and magnetic sensors available on commodity smartphones [1,2]. Recent PDR systems address attitude changes, magnetic disturbances, and three-dimensional motion through axis mapping, adaptive sensor fusion, and geomagnetic–barometric integration [4,5]. Within this broad application space, this work focuses on one specific and pervasive difficulty: the frequent changes of pedestrian action and phone carrying mode that repeatedly break the assumptions of a fixed step-length model. Localization is addressed in the two-dimensional (2D) horizontal plane, and segments involving vertical motion are outside the present scope. Deployments dominated by strong magnetic disturbance, underground structures, or multi-floor transitions remain future evaluation settings.

In step-based PDR, the horizontal position is updated by recursively combining the current step length and heading with the previous position estimate [1,2]. Because this integration is open loop, small systematic biases in step length or heading rapidly accumulate over long trajectories, leading to significant drift in real-world usage [1,2]. This motivates evaluations on long-distance routes where model mismatch and heading instability become observable at the trajectory level (see Section 4).

A major source of drift in practical smartphone PDR is model mismatch caused by diverse human motions and phone carrying modes [2,6,7]. Action types such as walking, jogging, and backward walking, together with carrying modes such as handheld, pocket, and arm-swinging, can drastically change inertial-signal statistics and the effective relationship between signal amplitude and true step length [6,7,8]. Recent gait-adaptive and personalized PDR systems explicitly model these variations through gait recognition, phone-location recognition, or state-dependent step-length estimation [9,10,11]. Conventional PDR approaches commonly assume a single step-length model with fixed parameters [1,2]. A representative example is the classical Weinberg model, which expresses step length as a scaled function of the peak-to-valley acceleration amplitude, with the proportionality factor *K* typically determined by offline calibration [1]. Once the user switches motion style or carrying mode, the assumed scale relationship becomes inconsistent, causing systematic over-/under-estimation of step length and accelerated error growth [6,7].

Existing adaptive step-length methods address this mismatch in two main ways. Mode-switching approaches recognize a small set of modes and switch to pre-calibrated parameters, which leaves within-mode variation and frequent state transitions insufficiently modeled [12,13]; more recent learning-based methods, including temporal deep models and deep-inertial odometry, largely replace the step-length model with a network, achieving high accuracy at the cost of interpretability and dependence on training coverage [14,15]. The specific gap addressed here is a step-length model that adapts continuously both across motion states and within a state while preserving the interpretable physical structure of the classical formulation.

This study therefore focuses on motion-state-aware adaptive step length modeling as a key mechanism for reducing model mismatch under frequent motion changes [6,7]. We introduce a joint motion state defined by the combination of action type and carrying mode, and use it as the conditioning variable for adaptive step-length parameterization [8]. Specifically, we build on the Weinberg model [1] but reinterpret its constant scale factor as a motion-conditioned coefficient. A state-wise linear model provides the main lightweight coefficient predictor, while a decoder-only Transformer [16] is used as a temporal enhancement to model short-term continuity in *K* from historical step features under distance-level offline supervision. This design preserves the physical structure of the Weinberg equation while replacing the fixed coefficient setting with adaptive models that capture state dependence and moderate within-state variability.

A joint motion-state recognition module operating on dynamic sliding windows of multi-sensor smartphone data provides the state priors used for conditional adaptation [8,17]. Motion recognition is not treated as an independent objective; rather, it supports the state-dependent components of the PDR model [6,7]. To improve the stability of heading estimation in magnetically disturbed GPS-denied environments, particularly indoor spaces, we combine gyroscope-based heading updates with magnetometer-based absolute observations through a Kalman-filter-based fusion scheme, while representing heading in a 2D unit-vector form in our implementation [18,19,20,21,22]. Such vector/quaternion representations are widely used in inertial–magnetic sensor fusion to avoid Euler-angle singularities and reduce discontinuities due to angle wrapping [20,22].

**Figure 1:**
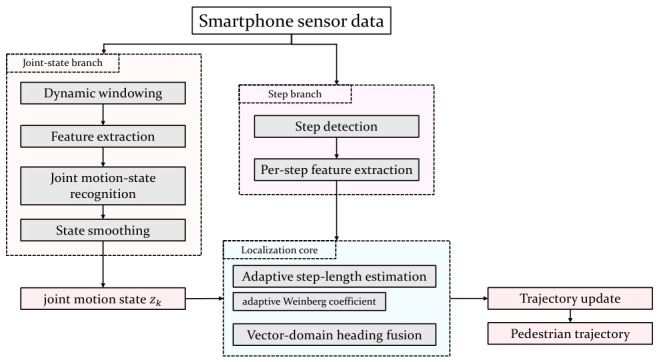
Proposed motion-state-aware PDR framework.

Figure 1 presents the proposed motion-state-aware extension with joint-state recognition, adaptive step-length modeling, and vector-domain heading fusion. Experiments on outdoor long routes and a representative indoor corridor, covering multiple motion types and carrying modes with frequent state transitions, are organized around coefficient-modeling diagnostics and trajectory-level localization evaluation under a shared PDR pipeline for planar pedestrian trajectories.

The main contributions of this paper are summarized as follows:

- **Continuous Weinberg-coefficient prediction with linear and Transformer-enhanced variants**: The paper introduces a joint motion state (action type × carrying mode), defines a state-wise linear model for lightweight coefficient adaptation, and evaluates a compact decoder-only Transformer as a temporal extension for estimating the per-step continuous coefficient *K* from historical step-feature sequences. Offline training uses segment-distance MSE, and the Weinberg step-length structure is preserved throughout the formulation.
- **Lightweight joint motion-state recognition for conditional adaptation**: A random-forest-based recognition module provides stable state priors to support conditional step-length adaptation without substantial computational overhead.
- **Vector-domain heading fusion**: Gyroscope–magnetometer fusion is performed in a unit-vector representation using Kalman filtering, yielding more stable trajectories in long-distance evaluations.

## 2. Related Work

### 2.1. Sensitivity of Smartphone PDR to Motion Conditions

Smartphone-based pedestrian dead reckoning (PDR) estimates a trajectory by iteratively combining step events, step length, and heading. Because these components are integrated in an open loop, small systematic biases (e.g., step-length mismatch or heading drift) accumulate with path length and quickly dominate the final error budget [1,2]. A persistent challenge is that the inertial signal statistics observed by a smartphone vary substantially across both locomotion types (e.g., level walking, running, stairs) and device carrying modes (e.g., handheld, pocket), causing classical fixed-parameter models to operate outside their calibration regime [2].

### 2.2. Step-Length Estimation Under Multiple Motions and Carrying Modes

Step-length estimation approaches can be broadly grouped into empirical models and data-driven regression models [1,2]. Parametric models remain attractive in mobile systems because they are simple to deploy and easy to interpret. A representative example is the Weinberg model, which relates step length to the fourth root of the peak-to-valley acceleration amplitude through a proportionality factor [1]. In most deployments, this factor is treated as a constant determined by offline calibration, which becomes brittle when motion type or carrying mode changes.

To improve robustness, several studies introduce context into step-length estimation. Early work used movement-status awareness and adaptive parameter selection such as walk/run switching [23], while smartphone-oriented systems further detect carrying modes and select corresponding model parameters in real time [12]. Recent methods combine multi-mode recognition with learned step-length estimation [9,10,13] or use motion-aware inertial-odometry networks with adaptive computational depth [15,24]. These approaches span discrete state selection, learned temporal regression, and end-to-end inertial odometry, with performance shaped by training coverage and cross-scenario conditions [2,25]. The present work retains the interpretable Weinberg structure, conditions its coefficient on the joint motion state, and adapts the coefficient continuously across and within joint states. The adaptive components highlighted in Figure 1 form a hybrid step-length model with an explicit physical backbone.

### 2.3. Motion and Device Pose Recognition for Adaptive PDR

Motion recognition and phone pose (carrying-mode) recognition are widely studied as front-end modules for adaptive localization. Mode-aware dead reckoning systems typically infer a small set of device modes and apply mode-specific step-length/heading strategies [12,13]. More recently, joint pedestrian motion state and device pose classification has been formulated as an IMU recognition problem [8], mixed-pose localization has been explored using clustering and pose-dependent updates [26], and smartphone PDR has been extended to attitude-unconstrained axis mapping and sensor fusion [4]. The present work uses a joint motion state (action × carrying mode) as a unified conditional variable for an interpretable step-length model with continuous within-state adaptation.

### 2.4. Heading Estimation Under Magnetic and Motion Disturbances

Heading estimation in pedestrian navigation combines short-term gyroscope continuity with an absolute or quasi-absolute reference. Recent PDR methods address magnetic and motion disturbances through complementary mechanisms. Adaptive gait detection and heading-drift correction combine period-dependent step detection with gyroscope bias compensation and trajectory correction [11]. Magnetic-interference-aware heading filters detect disturbances and adjust the filter response according to magnetometer reliability [27]. Deep-learning approaches classify magnetic disturbances or learn heading constraints from smartphone sensors, including CNN–DRL fusion-weight adaptation and visual straight-line cues [28,29]. Geomagnetic vector constraints and hybrid geomagnetic–PDR models provide additional environmental information for indoor trajectories [30,31]. These methods characterize the two principal sources of heading error considered in this work: inertial integration drift and disturbance of the magnetic observation.

### 2.5. Back-End Drift Suppression and Global Consistency

Beyond improving step-length and heading estimation, many systems suppress long-term drift by introducing constraints or global optimization, such as map matching, semantic-event corrections, zero-velocity updates (ZUPT) during stationary intervals, or factor-graph/SLAM-style back ends [1,2]. Map constraints alone can bound particle-filter drift without external position observations, while boundaries tightened beyond a scenario-dependent tolerance reduce accuracy [32]. Smartphone IMU-only SLAM methods that leverage repeated activities (e.g., turns) as loop-closure events can substantially constrain drift, at the expense of additional modeling and back-end computation [33]. These techniques operate on a different part of the pipeline than the present work and are complementary to it: the accuracy of any such back end is bounded by the quality of the front-end step-length and heading estimates it integrates, so reducing front-end model mismatch lowers the residual drift that a global corrector must absorb, and the two can be combined when maps or semantic cues are available.

### 2.6. Research Gap and Theoretical Positioning

In summary, existing approaches largely fall into four categories:

- **Fixed-parameter empirical models** (e.g., Weinberg) with strong interpretability but limited robustness under motion/pose changes [1];
- **Mode-switching PDR** that recognizes a small set of modes and selects pre-calibrated parameters, which can struggle under frequent state transitions and within-mode variability [12];
- **Fully data-driven regressors** that can be accurate on matched data but require extensive training coverage and provide limited physical interpretability [13,14,25];
- **Global optimization/SLAM back ends** that constrain drift but add algorithmic complexity and may rely on additional assumptions or cues [2,33].

Our approach is positioned between fixed empirical modeling, mode-switching adaptation, and fully data-driven regression. It retains the interpretable Weinberg structure, uses a joint state (action × carrying mode) to describe motion context, and predicts a continuous coefficient for feature-driven adaptation across states and within each state.

## 3. Methodology

### 3.1. Problem Setup and Overall Framework

Let $p_{k}={\left[x_{k},y_{k}\right]}^{⊤}$ denote the 2D pedestrian position in the world coordinate system W after the *k*-th valid step. The PDR pipeline estimates the trajectory ${\left\{p_{k}\right\}}_{k=0}^{K}$ from step length, heading, and motion-state information. Since open-loop PDR accumulates errors in step length and heading over time, we introduce a joint motion state as the conditioning variable:

(1) $$z_{t}=\left(a_{t},\;c_{t}\right),$$

where $a_{t}$ denotes the action category (e.g., walking, jogging, backward walking, and standing), and $c_{t}$ denotes the smartphone carrying mode (e.g., handheld, pocket, and arm-swinging). The joint motion state conditions step-length modeling while keeping the overall pipeline lightweight for smartphone deployment.

The system input consists of multi-sensor time series collected from a smartphone, including tri-axial acceleration $a\left(t\right)={\left[a_{x}\left(t\right),a_{y}\left(t\right),a_{z}\left(t\right)\right]}^{⊤}$, angular velocity $\omega \left(t\right)={\left[\omega_{x}\left(t\right),\omega_{y}\left(t\right),\omega_{z}\left(t\right)\right]}^{⊤}$, and magnetic field measurements $m\left(t\right)={\left[m_{x}\left(t\right),m_{y}\left(t\right),m_{z}\left(t\right)\right]}^{⊤}$, all expressed in the device coordinate frame B. To reduce sensitivity to device orientation changes and carrying modes, magnitude-based representations are used together with axis-wise signals:

(2) $$\begin{array}{cc} ∥a(t)∥ & =\sqrt{a_{x}^{2}\left(t\right)+a_{y}^{2}\left(t\right)+a_{z}^{2}\left(t\right)},\\ ∥\omega (t)∥ & =\sqrt{\omega_{x}^{2}\left(t\right)+\omega_{y}^{2}\left(t\right)+\omega_{z}^{2}\left(t\right)},\\ ∥m(t)∥ & =\sqrt{m_{x}^{2}\left(t\right)+m_{y}^{2}\left(t\right)+m_{z}^{2}\left(t\right)}. \end{array}$$

Raw acceleration, smoothed signals, and magnitude-based features are used together to balance robustness and discriminability across different motion patterns and carrying modes.

The proposed framework consists of four components. First, continuous sensor streams are segmented with overlapping windows, time- and frequency-domain features are extracted, and a supervised classifier estimates the joint motion state. Short-horizon smoothing stabilizes window-level predictions, and step timestamps are associated with the smoothed state to obtain step-level labels. Second, dynamic-threshold peak detection is applied to smoothed acceleration magnitude to obtain step timestamps and per-step descriptors such as period, frequency, amplitude, and energy. Third, the Weinberg model is retained as the step-length backbone, with a state-wise linear predictor and a decoder-only Transformer used to adapt the coefficient *K*. Fourth, gyroscope-based heading increments and magnetometer-based absolute heading observations are fused in the 2D vector domain using Kalman filtering.

### 3.2. Error Mechanisms Under Motion and Carrying-Mode Changes

The open-loop position update couples step-length and heading errors over the complete trajectory. Let $u\left(\theta_{k}\right)={\left[\cos\theta_{k},\sin\theta_{k}\right]}^{⊤}$ denote the unit displacement direction at step *k*, and let $\delta s_{k}$ and $\delta \theta_{k}$ denote small errors in step length and heading. A first-order expansion of the accumulated position error after *N* steps gives

(3) $$\delta p_{N}\approx \sum_{k=1}^{N}\left[u\left(\theta_{k}\right)\;\delta s_{k}+s_{k}u_{⊥}\left(\theta_{k}\right)\;\delta \theta_{k}\right],\;u_{⊥}\left(\theta_{k}\right)={\left[-\sin\theta_{k},\cos\theta_{k}\right]}^{⊤}.$$

Step-length errors contribute primarily along the direction of travel, while heading errors produce a cross-track component proportional to the step length. For approximately constant step length and cadence over a locally straight segment, a persistent step-length bias, $\delta s_{k}\approx \bar{\delta s}$, produces an O(N) along-track error. A constant effective heading-rate bias $b_{\theta }$ causes the open-loop heading error to grow approximately linearly with elapsed time, $\delta \theta_{k}\approx b_{\theta }k\Delta t$. Substitution into Equation (3) gives a cross-track term proportional to $\sum_{k=1}^{N}k$ and an $O(N^{2})$ worst-case growth over the interval in which the first-order approximation remains valid. The realized growth rates depend on route geometry and on the persistence and sign of the error terms; curvature and sign changes can produce partial cancellation [1,2]. In the proposed framework, joint-state conditioning reduces persistent step-length scale bias, and gyroscope–magnetometer fusion provides absolute heading observations that constrain angular drift.

For the Weinberg relation $s_{k}=K_{k}{\left(\Delta a_{k}\right)}^{1/4}$, small perturbations in the coefficient and peak-to-valley acceleration amplitude yield

(4) $$\frac{\delta s_{k}}{s_{k}}\approx \frac{\delta K_{k}}{K_{k}}+\frac{1}{4}\frac{\delta (\Delta a_{k})}{\Delta a_{k}}.$$

The coefficient $K_{k}$ represents the effective scale between the phone-observed acceleration pattern and pedestrian displacement. Locomotion type changes gait frequency, impact intensity, stance–swing timing, and the relationship between stride length and vertical body motion [6,10]. Carrying mode changes the measured inertial pattern because a handheld phone includes hand and forearm dynamics, a pocket constrains motion through contact with the body and clothing, and arm swinging introduces an additional periodic component [8]. These action- and carrying-dependent changes affect both $K_{k}$ and $\Delta a_{k}$. Accordingly, context-aware and personalized PDR methods condition step-length estimation on activity, phone location, or mobility context [7,9]. A coefficient calibrated for one action–carrying combination can therefore produce a systematic scale error after a state change.

Heading errors arise from a separate set of sensor mechanisms. The device-frame angular rate and acceleration can be written as

(5) $$\begin{array}{ccc} \omega^{B}\left(t\right) & = & R_{W}^{B}\left(t\right)\omega^{W}\left(t\right)+b_{g}\left(t\right)+\eta_{g}\left(t\right),\\ a^{B}\left(t\right) & = & R_{W}^{B}\left(t\right)\left(a_{\mathrm{lin}}^{W}\left(t\right)-g^{W}\right)+b_{a}\left(t\right)+\eta_{a}\left(t\right), \end{array}$$

where $R_{W}^{B}$ maps world-frame quantities into the device frame, $a_{\mathrm{lin}}^{W}$ is non-gravitational acceleration, and $g^{W}={\left[0,0,-g\right]}^{⊤}$ is gravitational acceleration in a world frame whose vertical axis points upward. The accelerometer therefore senses specific force, and a stationary device reports a vertical component of magnitude *g* along its upward axis. The terms $b_{g}$ and $b_{a}$ denote gyroscope and accelerometer biases, and $\eta_{g}$ and $\eta_{a}$ denote sensor noise. Gyroscope bias accumulates during integration, while vigorous motion increases $a_{\mathrm{lin}}^{W}$ and degrades acceleration-based roll and pitch estimates [20,21]. Accelerometer bias tilts the estimated gravity direction and introduces a systematic roll and pitch offset, which carries into the vertical angular rate obtained from the device-frame measurement. Phone reorientation changes the device-to-world mapping used for that extraction [4]. The magnetometer supplies an absolute heading reference, but nearby ferromagnetic structures and electrical equipment perturb the local magnetic field and can bias the resulting observation [27,28,29]. Motion and carrying mode primarily affect body–device kinematics and attitude estimation; magnetic disturbance is an environmental contribution to heading error.

These mechanisms define the roles of the adaptive components in the framework. Joint-state conditioning accounts for action- and carrying-dependent scale changes in Equation (4), and per-step descriptors capture variation within each joint state. Temporal coefficient modeling regularizes changes across consecutive steps and state transitions. Vector-domain gyroscope–magnetometer fusion combines short-term angular-rate continuity with an absolute magnetic observation for the heading term in Equation (3).

### 3.3. Action–Carry Joint Motion-State Recognition

#### 3.3.1. Dynamic Windowing and Preprocessing

Let the sampling frequency be $f_{s}$. We segment the multi-sensor streams using overlapping sliding windows with length $N_{w}$ samples and step size $N_{s}$ samples. Each window contains tri-axial acceleration, angular velocity, and magnetic field measurements.

To reduce high-frequency noise while preserving gait periodicity, we apply smoothing (moving average followed by Savitzky–Golay filtering) on each channel [34]. The recognition pipeline uses overlapping windows together with short-horizon temporal smoothing to stabilize joint-state estimates.

Magnitude signals are computed for acceleration, angular velocity, and magnetic field to reduce sensitivity to device orientation changes. Raw acceleration, axis-wise signals, and magnitudes are used together for recognition and step analysis.

#### 3.3.2. Feature Extraction and Joint Label Definition

For each window, we extract features from the filtered acceleration, angular-velocity, and magnetic-field channels and their magnitude signals. Representative features include mean, variance, RMS, skewness, peak–valley count, Shannon entropy, PSD mean, and band-energy descriptors. In total, 24 features are extracted per channel, resulting in a 288-dimensional feature vector for the 12 channels used in this work. The joint motion state is defined as the Cartesian product of an action set A and a carrying-mode set C:

(6) Z=A×C,z=(a,c)∈Z.

The recognition task is formulated as multi-class classification over Z.

#### 3.3.3. Classifier and Temporal Smoothing

A Random Forest (RF) classifier maps window features to joint-state predictions [35]. To suppress boundary-window jitter and transient misclassifications, we apply a short-horizon temporal smoothing strategy (e.g., majority voting over the latest *L* window predictions) to obtain a stable joint-state prior $\tilde{z}\left(t\right)$. This smoothed state is subsequently associated with step events to produce a step-level label $z_{k}$.

### 3.4. Step Detection and Per-Step Feature Extraction

#### 3.4.1. Smoothed Acceleration Magnitude for Peak Detection

Step events are detected from a step-sensitive signal, chosen as the smoothed acceleration magnitude (denoted as $a_{m}\left(t\right)$) [1,2]. Candidate steps are identified by peak detection under two constraints: (i) a minimum peak-to-peak time interval to ensure physically plausible step frequency, and (ii) a minimum peak–valley amplitude to reject spurious peaks.

#### 3.4.2. Dynamic Thresholding

To adapt to amplitude variations caused by different motions and carrying modes, we use a dynamic threshold defined by local statistics:

(7) $$\tau =T_{0}+\alpha \left(\mu_{v}+\sigma_{v}\right),\;\tau \leq \tau_{\max},$$

where $\mu_{v}$ and $\sigma_{v}$ are computed over a short history of recent peak–valley differences, $T_{0}$ is a base threshold, and α controls adaptivity. A minimum peak–valley difference $v_{\min}$ is also enforced.

Let $\left\{t_{k}\right\}$ be the detected step timestamps. For each step, we compute the step period $\Delta t_{k}=t_{k}-t_{k-1}$ and step frequency $f_{k}=1/\Delta t_{k}$, which are used both for step length estimation and for characterizing motion dynamics.

### 3.5. Motion-State-Conditioned Step Length Estimation with a Decoder-Only Transformer

#### 3.5.1. Weinberg Baseline

We adopt the Weinberg model as an interpretable baseline [1]:

(8) $$s_{k}=K\cdot {\left(\Delta a_{k}\right)}^{\frac{1}{4}},\;\Delta a_{k}=a_{k}^{\max}-a_{k}^{\min},$$

where $a_{k}^{\max}$ and $a_{k}^{\min}$ are the maximum and minimum values of the step-sensitive signal within the *k*-th step interval, and *K* is a scale factor.

#### 3.5.2. State-Wise Linear Coefficient Prediction

To provide a lightweight adaptive coefficient model, we first define a state-wise linear variant in which the scale factor is conditioned on the step-level joint state $z_{k}$ and a per-step feature vector $u_{k}$:

(9) $$s_{k}=K_{k}\cdot {\left(\Delta a_{k}\right)}^{\frac{1}{4}}.$$

The linear coefficient predictor is written as

(10) $$K_{k}^{\mathrm{lin}}=\max\;\left(0,\alpha_{0,z_{k}}+\beta_{z_{k}}^{⊤}u_{k}\right),$$

where $\alpha_{0,z}$ is a state-specific offset term, $\beta_{z}$ is a state-specific correction vector, and the maximum operator avoids invalid negative scale values. This non-negativity constraint applies only to the step-length scale. Motion states such as backward walking and standing are handled through a state-dependent displacement factor in the position update.

In this implementation, $u_{k}$ includes frequency/period-related and amplitude/energy-related step descriptors:

(11) $$u_{k}={\left[\;f_{k},\;\Delta t_{k},\;\Delta f_{k},\;{\mathrm{cv}}_{t,k},\;{\mathrm{rms}}_{k},\;{\mathrm{rpv}}_{k},\;e_{k}^{\mathrm{unit}},\;{\mathrm{cv}}_{\Delta a,k},\;{\mathrm{cv}}_{\mathrm{rms},k}\;\right]}^{⊤},$$

where $\Delta f_{k}=f_{k}-f_{k-1}$ denotes the first-order variation of consecutive step frequencies, ${\mathrm{cv}}_{t,k}$ denotes the coefficient of variation of recent step periods, ${\mathrm{rms}}_{k}$ is the root mean square of the raw acceleration signal within the *k*-th step interval, ${\mathrm{rpv}}_{k}=a_{k}^{\max}/\left(\right|a_{k}^{\min}\left|+\varepsilon \right)$ is the peak-to-valley ratio, and $e_{k}^{\mathrm{unit}}=\mathrm{mean}\left(a^{2}\right)/\Delta t_{k}$ is an energy-related descriptor normalized by step duration. Here, ${\mathrm{cv}}_{\Delta a,k}$ and ${\mathrm{cv}}_{\mathrm{rms},k}$ denote the coefficients of variation of recent peak-to-valley amplitudes and RMS values, respectively, computed over a short sliding window. The vector $u_{k}$ represents the per-step descriptor set shared by both the linear and Transformer-enhanced variants.

#### 3.5.3. Decoder-Only Transformer for Continuous-Value Prediction of *K*

The Transformer-enhanced variant is designed as a compact temporal extension to the state-aware coefficient model. It predicts the continuous coefficient $K_{k}$ with a lightweight decoder-only Transformer [16] operating on the historical step sequence. For each step *k*, the model outputs one coefficient estimate, and the resulting step-length sequence is trained through the accumulated distance error of each calibration segment. The input token is formed from the step-level joint-state embedding and the numerical descriptors:

(12) $$x_{k}={\left[e{\left(z_{k}\right)}^{⊤},u_{k}^{⊤},\Delta a_{k}\right]}^{⊤},$$

where $e(z_{k})$ denotes the learnable embedding of the joint motion state. The token representation is projected to the model dimension and combined with positional information:

(13) $$r_{k}=W_{\mathrm{in}}x_{k}+b_{\mathrm{in}}+p_{k}.$$

Given the sequence $\left(r_{1},r_{2},\ldots ,r_{k}\right)$, the decoder-only Transformer applies masked self-attention so that the representation at step *k* depends only on the current and previous steps:

(14) $$\left(h_{1},h_{2},\ldots ,h_{k}\right)=\mathrm{TransformerDecoder}\left(r_{1},r_{2},\ldots ,r_{k};M\right),$$

where M denotes the causal attention mask. The continuous coefficient is predicted from the final hidden state through a regression head:

(15) $${\hat{K}}_{k}^{\mathrm{tr}}=\mathrm{softplus}\;\left(w_{o}^{⊤}h_{k}+b_{o}\right).$$

The predicted coefficient is then inserted into the Weinberg equation:

(16) $${\hat{s}}_{k}={\hat{K}}_{k}^{\mathrm{tr}}\cdot {\left(\Delta a_{k}\right)}^{\frac{1}{4}}.$$

In implementation, the decoder follows a standard causal Transformer design with rotary positional encoding (RoPE) [36], RMSNorm [37], and SwiGLU-style feed-forward blocks [38]. The equations above focus on the coefficient-prediction path used for step-length estimation. This formulation preserves the empirical structure of the step-length model while adding a small temporal modeling component over historical step features.

#### 3.5.4. Offline Training

The linear-variant parameters $\left\{\alpha_{0,z},\beta_{z}\right\}$ are obtained by an offline closed-form least-squares fit to the segment distances of the controlled calibration segments, and serve as the low-complexity adaptive model. The operator max(0,·) clips the predicted step-length scale to non-negative values at prediction time. The learnable joint-state embedding $e(z_{k})$ of the Transformer variant and the state-specific parameters $\left\{\alpha_{0,z},\beta_{z}\right\}$ of the linear variant are separate parameter sets. During offline training, the Transformer uses the same calibration segments and predicts one coefficient for each step. For a calibration segment containing *N* detected steps with known reference distance $D^{*}$, the predicted total distance is obtained by summing the step-wise distances defined above:

(17) $$\hat{D}=\sum_{k=1}^{N}{\hat{s}}_{k}=\sum_{k=1}^{N}{\hat{K}}_{k}^{\mathrm{tr}}\cdot {\left(\Delta a_{k}\right)}^{\frac{1}{4}}.$$

The training objective then minimizes the mean squared error between the predicted segment distance and the calibrated reference distance:

(18) $$L_{D}=\frac{1}{M}\sum_{m=1}^{M}{\left({\hat{D}}_{m}-D_{m}^{*}\right)}^{2},$$

where *M* is the number of calibration segments in the training set. Each step contributes through its predicted coefficient ${\hat{K}}_{k}^{\mathrm{tr}}$, and the optimization target is the accumulated distance consistency over the full labeled segment. The input sequence used during training and inference is the historical feature sequence up to the current time step, so the prediction of ${\hat{K}}_{k}^{\mathrm{tr}}$ is causal. Online deployment uses the same step detector and feature extractor as the linear variant, and the two models differ only in the mapping from historical features to the coefficient $K_{k}$. Steps detected during a standing state are kept in the input sequence, which preserves the continuity of the temporal context. For a standing step the displacement factor $\gamma (z_{k})=0$ in the position update, so its coefficient does not affect the trajectory.

### 3.6. Vector-Domain Heading Fusion

#### 3.6.1. Heading Representation

To avoid discontinuities caused by angle wrapping at ±π, we represent heading using a 2D unit vector. Related non-Euler vector/quaternion representations are widely used in orientation estimation and inertial–magnetic fusion [19,20,21,22]:

(19) $$h_{k}=\left[\begin{array}{c} \cos\theta_{k}\\ \sin\theta_{k} \end{array}\right].$$

#### 3.6.2. Gyroscope-Based Prediction

Between two consecutive steps, the heading increment $\Delta \theta_{k}$ is predicted from the component of angular velocity aligned with the vertical axis of the world frame. Since the smartphone may undergo pose changes across different carrying modes, the measured angular velocity $\omega^{B}\left(t\right)$ in the device frame is related to the world frame through the current attitude estimate, and the corresponding vertical-axis component is integrated over the step interval:

(20) $$\Delta \theta_{k}=\int_{t_{k-1}}^{t_{k}}\omega_{z}^{W}\left(t\right)\;dt.$$

The predicted heading vector is

(21) $$h_{k}^{-}=R\left(\Delta \theta_{k}\right)\;h_{k-1},$$

where $\omega_{z}^{W}\left(t\right)$ denotes the vertical-axis angular-rate component in the world frame and R(·) is the 2D rotation matrix. In this way, gyroscope-based updating is defined with respect to planar heading change in the world frame.

#### 3.6.3. Magnetometer Observation (Tilt-Compensated)

The magnetometer provides an absolute heading observation. We compute a tilt-compensated yaw by first estimating pitch and roll from acceleration, and then combining pitch/roll with magnetometer measurements to obtain $\theta_{k}^{m}$. The corresponding observation is represented as

(22) $$z_{k}=\left[\begin{array}{c} \cos\theta_{k}^{m}\\ \sin\theta_{k}^{m} \end{array}\right].$$

#### 3.6.4. Vector-Domain Kalman Update

We fuse $h_{k}^{-}$ and $z_{k}$ using a Kalman-filter-based heading update. The vector-domain form adopted here is our implementation choice, motivated by prior Kalman-based heading fusion and non-Euler attitude representations [18,20,21]. Let $P_{k}$ denote the covariance of $h_{k}$. With diagonal process noise Q and measurement noise R, the update is

(23) $$K_{k}=P_{k}^{-}{\left(P_{k}^{-}+R\right)}^{-1},\;h_{k}=h_{k}^{-}+K_{k}\left(z_{k}-h_{k}^{-}\right),$$

followed by normalization $h_{k}\leftarrow h_{k}/∥h_{k}∥$. The matrices Q, R, and the initial covariance $P_{0}$ are diagonal and are selected empirically, guided by typical process-to-measurement noise ratios for gyroscope–magnetometer fusion, and then kept unchanged throughout the evaluation.

### 3.7. Position Update and Motion-State Association

Given step length $s_{k}$ and heading vector $h_{k}$, the 2D position is updated by

(24) $$p_{k}=p_{k-1}+\gamma \left(z_{k}\right)\;s_{k}\;h_{k}.$$

Here, the state-dependent displacement factor is defined as

(25) $$\gamma \left(z_{k}\right)=\left\{\begin{array}{cc} -1, & \mathrm{if}\;z_{k}\;\mathrm{corresponds}\;\mathrm{to}\;\mathrm{backward}\;\mathrm{walking},\\ 0, & \mathrm{if}\;z_{k}\;\mathrm{corresponds}\;\mathrm{to}\;\mathrm{standing},\\ 1, & \mathrm{otherwise}. \end{array}\right.$$

The joint motion state $z_{k}$ is therefore used both to select the corresponding step-length parameters $\left\{\alpha_{0,z_{k}},\beta_{z_{k}}\right\}$ and to determine the displacement direction convention in the position update. Setting γ=0 for the standing state holds the position fixed while the pedestrian is stationary, which provides a zero-velocity behavior at the step level and prevents drift from accumulating during standing intervals.

## 4. Experiments

### 4.1. Experimental Setup

#### 4.1.1. Participants and Devices

Experiments were conducted with 23 participants (15 male and 8 female), with heights ranging from 159 cm to 189 cm, to evaluate the proposed motion-state-aware PDR framework under realistic walking conditions. Each participant contributed approximately 1.3 km of walking across the different motion types and carrying modes, amounting to about 30 km in total. All results are obtained under a subject-disjoint 7:3 train–test split, with 16 participants used for training and 7 held out for testing; no per-user calibration is applied at test time.

Data were collected using consumer-grade smartphones, including an iPhone 14 Pro (Apple Inc., Cupertino, CA, USA) running iOS 26.1 and an iPhone XR (Apple Inc., Cupertino, CA, USA) running iOS 18.7. The built-in tri-axial accelerometer, gyroscope, and magnetometer were used. All sensors were sampled at a fixed frequency of 50 Hz, and the recorded data streams were resampled to this common rate to ensure temporal alignment across sensors. The framework relies only on the inertial and magnetic sensors that are standard on commodity smartphones and on amplitude- and timing-based signal statistics, without dependence on platform-specific sensor-fusion services or device-specific calibration, so the pipeline is not tied to a particular hardware platform.

Before data collection, magnetometers were calibrated using standard hard-iron and soft-iron calibration procedures, including figure-eight motions, to reduce systematic magnetic bias.

**Figure 2:**
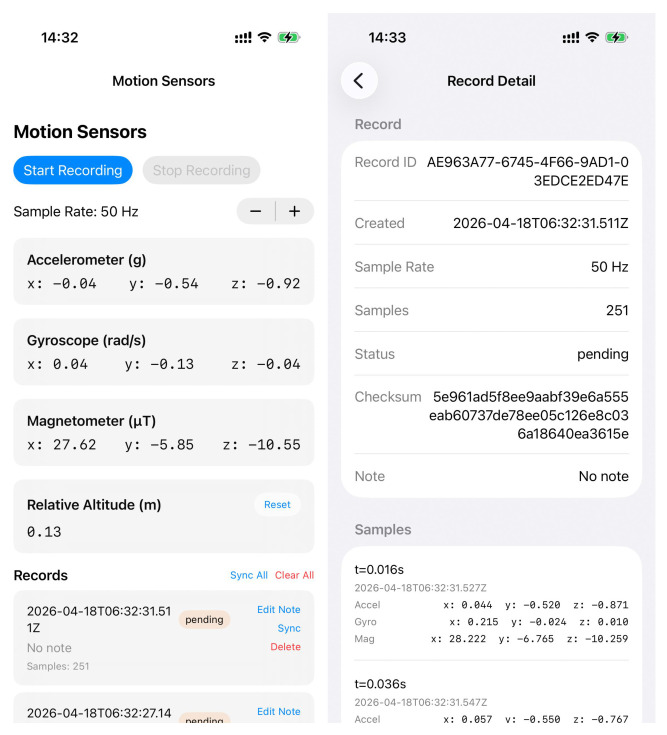
Screenshots of the smartphone application used for sensor-data collection in the experiments. The sensor readings displayed in the screenshots correspond to an uncalibrated state.

Sensor data were recorded using a custom-developed iOS application that has no formal version number and is not publicly distributed. Figure 2 shows the application used for synchronized collection of accelerometer, gyroscope, and magnetometer data during the experiments.

#### 4.1.2. Motion Types and Carrying Modes

To reflect realistic usage scenarios, multiple pedestrian motion types and smartphone carrying modes were considered. The motion types in the experiments were standing, walking, jogging, and backward walking. The smartphone carrying modes were handheld, pocket, and arm-swinging.

The joint-state labels used to train the recognizer were specified manually. Before each recording, the motion type and carrying mode to be performed were assigned in advance, and the participant switched to the designated motion at pre-agreed time points during the walk by monitoring the elapsed time. Labels are therefore defined at the level of time intervals within a recording rather than annotated frame by frame, and they share the same clock as the 50 Hz sensor stream recorded by the collection application, so no separate post hoc alignment is required. Small timing offsets can occur around the transition instants between two motions; because each recording is relatively long, these transition intervals account for only a small fraction of the total data.

A joint motion state is defined as the combination of motion type and carrying mode. All experiments and evaluations are conducted in planar (2D) space. Data segments involving vertical movement are excluded from analysis.

Table 1 summarizes the main experimental conditions and the joint motion-state categories considered in this study.

#### 4.1.3. Training and Testing Routes

Two types of routes were designed for training and evaluation:

(1) **Controlled calibration routes**: Each participant completed multiple controlled 50 m and 100 m calibration segments while performing different combinations of motion types and carrying modes, so that every joint motion state was covered. These data were used for joint motion state recognition training and step-length-model calibration. Each segment was treated as one calibration unit with known reference distance equal to the route length. The detected steps within the segment were used to construct the per-step feature sequence, and the known segment distance was used as the supervision signal for segment-level distance fitting. The average step length of a segment was obtained by dividing the known route length by the number of detected steps and was used as a descriptive calibration statistic. Across all participants, the collected data amount to approximately 30 km of walking and on the order of $5\times 10^{4}$ detected steps.

(2) **Testing routes**: Three representative testing scenarios were considered:

- A 400 m outdoor running track, where each participant completed one full lap (a few participants two laps) under different carrying modes and motion types.
- A circular route, completed once per participant, used to examine trajectory consistency on closed-loop motion.
- An indoor corridor of approximately 271 m, completed by a subset of 4 participants (one run each). The route contains multiple right-angle turns and passes near structural steel and electrical rooms, so it carries realistic building magnetic disturbance in addition to the motion-state transitions.

Motion type and carrying mode transitions were performed at predefined route nodes. On average, each testing route contained approximately three state transitions.

#### 4.1.4. Ground Truth and Error Evaluation

Ground truth trajectories were obtained using manually annotated reference points combined with tape-measured distances. All estimated and reference trajectories are expressed in a local 2D Cartesian coordinate frame with the origin at the route start, and reference points were placed at an interval of approximately 0.5 m along each route. The resulting reference polyline provides a practical route-level benchmark for evaluating trajectory drift.

Localization error was evaluated in planar space. For each estimated trajectory, the point-wise localization error was computed as the shortest Euclidean distance from the estimated position to the reference trajectory polyline. This distance was used as the instantaneous localization error for performance evaluation.

#### 4.1.5. Transformer Evaluation Setting

For coefficient prediction, the Transformer was used as a lightweight causal sequence regressor over the historical per-step feature stream. The decoder used 2 layers, model dimension 8, 2 attention heads, and feed-forward dimension 16. The input token at each step consisted of the joint-state embedding together with the numerical descriptors defined in Section 3.5, and the current coefficient estimate was produced through a scalar regression head. Offline training was conducted on the controlled calibration trials, with each trial treated as one labeled calibration segment. The predicted step lengths were summed within the segment, and the segment distance was optimized with mean squared error against the known route length. Standard decoder components, including causal self-attention [16], rotary positional encoding (RoPE) [36], RMSNorm [37], and SwiGLU-style feed-forward blocks [38], were adopted directly. Under this lightweight configuration, the resulting model size is approximately $1.5\times 10^{3}$ trainable parameters.

The model was trained with the AdamW optimizer using a learning rate of $1\times 10^{-3}$, a weight decay of $1\times 10^{-4}$, and the segment-distance mean-squared-error objective, for 200 epochs with a fixed random seed. Each calibration segment was treated as a single training sequence whose length equals the number of steps detected in that segment, and the model parameters were updated once per segment (a batch size of one sequence); the segments were processed sequentially within each epoch. No dropout, learning-rate scheduling, or early stopping was used, and the input features were standardized before training.

Data preprocessing was performed in Python 3.11.11 using NumPy 2.4.0 and SciPy 1.16.3. The Random Forest classifier was implemented using scikit-learn 1.8.0, and the Transformer model was implemented using PyTorch 2.12.0.

### 4.2. Joint Motion State Recognition Results

**Figure 3:**
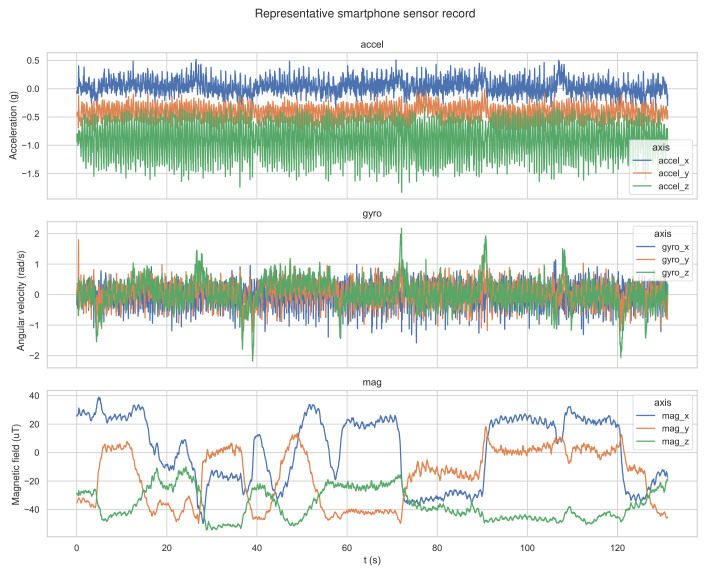
Example raw multi-sensor recordings collected by the smartphone (acceleration, angular velocity, and magnetic field). The signal characteristics motivate windowed processing and lightweight smoothing.

**Figure 4:**
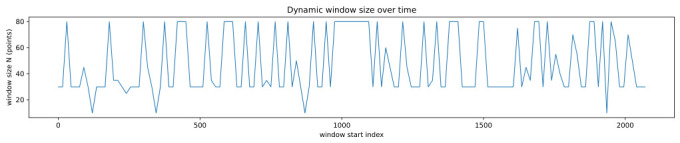
Sliding-window configuration for joint motion-state recognition (window length and stride).

Joint motion state recognition was implemented using a Random Forest classifier with dynamic sliding windows. The window length was set to 30 samples, with a step size of 15 samples. Feature extraction was performed on 12 normalized and filtered sensor channels, including acceleration, angular velocity, and magnetic field components and their magnitudes. A total of 288 features were extracted per window, including both time-domain and frequency-domain features. Figure 3 shows a representative excerpt of the raw multi-sensor recordings used in this stage, while Figure 4 illustrates the sliding-window configuration.

The Random Forest classifier consisted of 240 decision trees with a maximum depth of 15, and a minimum of 10 samples per leaf node. The classifier was evaluated using a subject-disjoint 7:3 train–test split: the 23 participants were divided into 16 training participants and 7 held-out testing participants before windows were generated. All recordings and overlapping windows from a given participant were assigned entirely to one subset, so no participant appeared in both training and testing. The recognition and localization results are thus reported under a subject-independent protocol. During training, class balancing was applied to reduce the influence of uneven sample counts among joint motion states.

Under this subject-disjoint evaluation protocol, the classifier achieved stable recognition performance across the considered joint motion states. Table 2 reports the per-class precision, recall, and F1-score, together with aggregate accuracy and averaged scores. Most walking states reached F1-scores above 0.93, while running and backward-walking cases showed larger variation because their dynamic patterns are closer near transition boundaries. The standing states also remained stable, although their scores were not perfect because natural phone use during standing can still introduce hand motion, posture adjustment, and small body sway in the sensor signals.

### 4.3. Step Characteristics and Coefficient-Prediction Diagnostics

In this section, *state* refers to the joint motion state defined by motion type and carrying mode. The complete state set contains 12 combinations formed by four motion types and three carrying modes. Since the standing state does not produce a valid displacement update, step-length estimation is effectively applied to the remaining nine active motion states. In the proposed pipeline, window-level recognition results are temporally smoothed and then associated with detected step events to obtain a step-level state label for each step. This label is used together with the step-related descriptors to construct the per-step input sequence for coefficient prediction.

**Figure 5:**
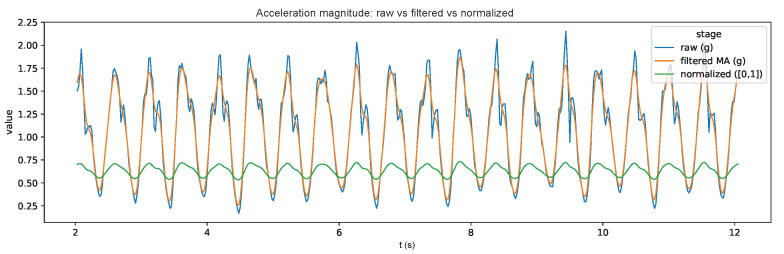
Example preprocessing of acceleration magnitude for enhancing step-related periodic patterns and suppressing high-frequency noise.

**Figure 6:**
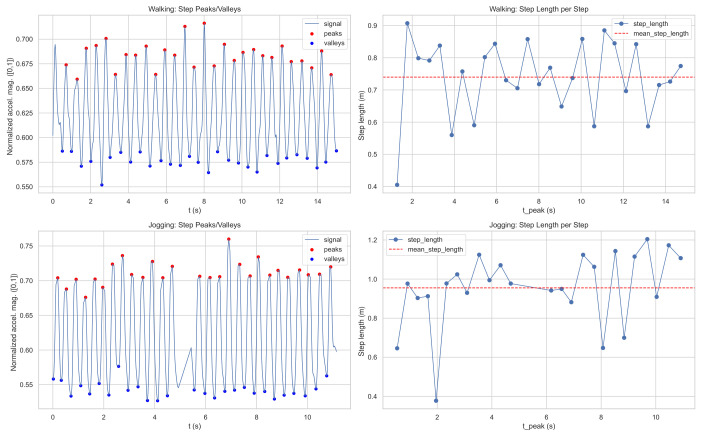
Representative handheld walking and jogging examples showing peak–valley detection and the resulting per-step step-length estimates.

To better understand how motion states and carrying modes affect step dynamics, we analyze step-frequency and step-length statistics derived from the same online step detector used in deployment. Figure 5 presents the preprocessed acceleration-magnitude signal used for step detection. Figure 6 presents two representative handheld examples, one walking and one jogging, to illustrate how peak–valley detection, state labeling, and coefficient prediction lead to the final per-step step-length estimates.

We further visualize the absolute errors of coefficient-based step-length modeling to compare the fixed-parameter baseline, the proposed linear variant, and the Transformer-enhanced variant.

**Figure 7:**
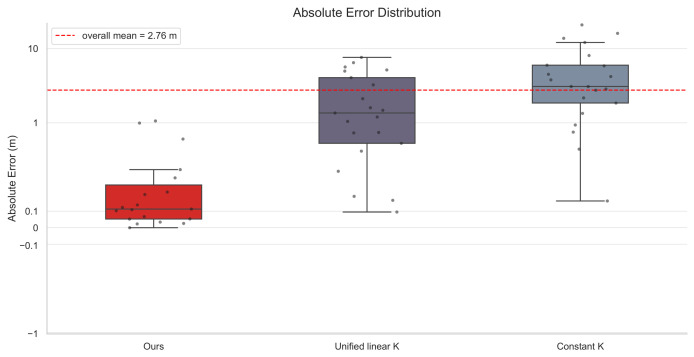
Absolute error analysis of step-length modeling, reflecting the impact of motion-state conditioning. Gray dots denote the individual absolute-error observations used to construct each boxplot.

Figure 7 summarizes the absolute-error distribution of the three main coefficient-prediction settings: the fixed-parameter model as the conventional reference, the state-wise linear model as the low-complexity adaptive variant, and the decoder-only Transformer as the temporal enhancement. These settings use the same detected steps and the same Weinberg step-length equation, so the observed differences are attributable to the coefficient-prediction module.

To further separate step-detection errors from coefficient-modeling errors, we assess the step detector itself. On a randomly sampled subset of recordings with manually verified step references, the detector achieves a miss rate of approximately 2.4% and a false-positive rate of approximately 3.3%, so step events are reliably detected across the joint states.

### 4.4. Localization Performance Comparison

Localization performance is evaluated by comparing six step-length settings: (i) a conventional smartphone PDR baseline with a fixed empirical Weinberg coefficient, (ii) a frequency–variance adaptive step-length baseline [23], (iii) a mode-switching Weinberg baseline [12,13], (iv) the proposed state-wise linear coefficient model, (v) a first-order exponential moving average (EMA) of the linear coefficient, and (vi) the Transformer-enhanced coefficient model. All six settings operate on the same detected steps and the same vector-domain heading fusion output, and differ only in the step-length model, so the comparison isolates step-length estimation from step detection and heading estimation. The evaluation metrics include mean trajectory error (MTE), root mean square error (RMSE), and distance-normalized error (Norm. Error), defined as $\mathrm{Norm}.\;\mathrm{Error}=100\times \mathrm{MTE}/D_{\mathrm{ref}}\;\%$, where $D_{\mathrm{ref}}$ is the tape-measured reference length of the route: 400 m for the running track, 74 m for the circular route, and 271 m for the indoor corridor.

**Figure 8:**
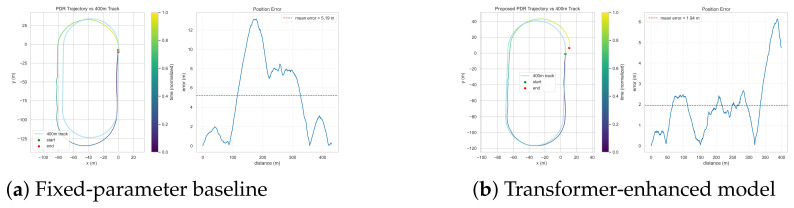
Trajectory comparison on the 400 m running-track route. (**a**): Conventional fixed-parameter baseline. (**b**): Transformer-enhanced coefficient model.

**Figure 9:**
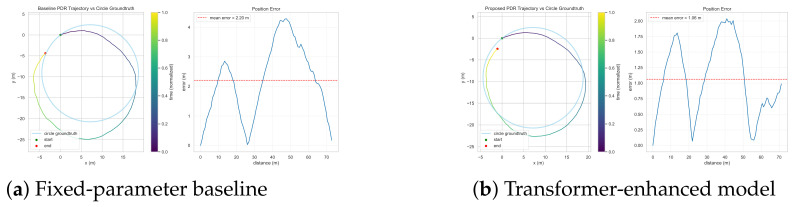
Trajectory comparison on the circular route. (**a**): Conventional fixed-parameter baseline. (**b**): Transformer-enhanced coefficient model.

**Figure 10:**
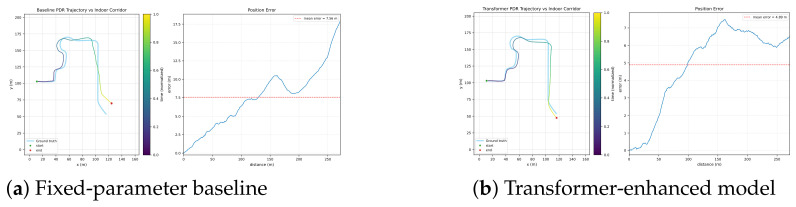
Trajectory comparison on the indoor corridor route. (**a**): Conventional fixed-parameter baseline. (**b**): Transformer-enhanced coefficient model. The corridor includes right-angle turns and building magnetic disturbance.

Representative trajectory panels are organized in Figure 8, Figure 9 and Figure 10. In each figure, the left panel shows the fixed-parameter baseline and the right panel shows the Transformer-enhanced coefficient model on the same route.

Quantitative evaluation is conducted with mean trajectory error (MTE), root mean square error (RMSE), and distance-normalized error. Table 3 reports the route-level metrics for the fixed-parameter baseline, the two adaptive step-length baselines used as reference points, the proposed state-wise linear model, a first-order exponential moving average (EMA) of the linear coefficient, and the Transformer-enhanced model. The EMA variant applies a smoothing factor of a=0.32 to the per-step coefficient produced by the linear model, serving as a lightweight temporal-smoothing reference against which the Transformer is compared. The trajectory panels in Figure 8, Figure 9 and Figure 10 visualize the fixed baseline and the final temporal model, while the table additionally includes the linear and EMA variants to show the effect of state-wise coefficient adaptation and simple smoothing before causal sequence modeling is introduced.

The long-route evaluation emphasizes cumulative localization behavior under repeated state transitions. The fixed-parameter baseline reflects the effect of using a constant coefficient, the proposed linear variant reflects joint-state-conditioned coefficient adaptation, and the Transformer-enhanced variant evaluates whether a compact causal sequence model can provide additional temporal regularization of the coefficient trajectory.

On the 400 m running-track route, the main improvement appears in the suppression of cumulative drift. The transition from the fixed baseline to the state-wise linear variant accounts for most of the error reduction, showing that joint-state-conditioned coefficient adaptation is important for long-distance walking with repeated motion changes. Simple EMA smoothing of the linear coefficient recovers much of the remaining gain on this route (0.48%), and the Transformer-enhanced model reaches 0.47%. On a route dominated by long straight segments, the difference between simple smoothing and causal sequence modeling is therefore small, indicating that temporal regularization of the coefficient sequence is useful but secondary to state-wise adaptation.

On the circular route, the improvement is reflected more strongly in trajectory shape consistency. The adaptive models reduce both the average deviation from the reference path and the loop-closure mismatch, with the Transformer-enhanced model reaching the lowest normalized error of 1.86%. Here the two temporal approaches separate: first-order exponential moving average smoothing yields 1.99%, essentially matching the linear variant (2.00%), whereas on the straight-dominated 400 m route it recovered most of the remaining gain. This indicates that when the heading changes continuously along the route, causal sequence modeling captures short-term coefficient dynamics that first-order smoothing does not, which is consistent with the improved curvature tracking in Figure 9.

The indoor corridor is the most demanding of the three routes, and was collected from a subset of 4 participants. Absolute errors are larger than on the outdoor routes because the corridor combines right-angle turns with building magnetic disturbance from nearby structural steel and electrical rooms, which perturbs the magnetometer observations that stabilize heading. Under these conditions the fixed-parameter baseline drifts to a normalized error of 2.79%, and state-wise coefficient adaptation reduces this to 2.22%. Temporal smoothing of the coefficient yields the largest relative improvement on this route, with the EMA and Transformer variants reaching 1.80% and 1.79% respectively. The two temporal approaches are effectively tied here: unlike the circular route, where continuous heading change favors causal sequence modeling, the corridor is dominated by discrete turns separated by straight segments, so the first-order exponential moving average captures most of the available temporal regularization. The corridor trajectories in Figure 10 show that the adaptive model tracks the reference path and its turns markedly more closely than the baseline, confirming that the framework remains effective in an indoor GPS-denied setting with realistic magnetic disturbance.

### 4.5. Comparison with Published Step-Length Models

Two adaptive step-length baselines representing the established strategies in the literature are evaluated in the same pipeline as reference points. The first follows the frequency–variance formulation, in which step length is a linear combination of a constant, step frequency, and acceleration variance, with a separate parameter set for each movement status [23]. The second follows the mode-switching strategy used in smartphone multi-mode PDR, in which a constant Weinberg coefficient is selected according to the recognized carrying mode [12,13]. Both are calibrated on the training participants only and are not recalibrated at test time, and both operate on the same detected steps and the same heading estimates as the proposed variants, so the comparison reflects the step-length model alone.

Both baselines improve substantially on the fixed-coefficient baseline, which confirms that parameter adaptation is the dominant factor on routes with repeated motion changes. Of the two, the mode-switching baseline is the more accurate on every route: conditioning on movement status alone leaves the carrying-mode component of the scale error unmodelled, and both factors change during the experiments.

The proposed state-wise linear model gives a lower normalized error than both baselines on all three routes. The margin is widest on the 400 m track, where repeated action changes over a long distance let a single mode-dependent constant accumulate scale error, and narrowest in the corridor, where heading disturbance contributes a larger share of the total error for every method. Temporal coefficient modeling adds a further reduction, and the Transformer-enhanced model gives the lowest normalized error on all three routes, approximately 38%, 13%, and 22% below the mode-switching baseline; relative to the state-wise linear model alone, temporal modeling contributes most in the corridor.

These results separate the contribution of each component. Conditioning the coefficient jointly on action type and carrying mode improves on single-factor parameter selection, and modeling the coefficient sequence across consecutive steps and state transitions produces the remaining reduction.

### 4.6. Joint-State vs. Single-Factor Ablation

The joint motion state combines an action type and a carrying mode into a single conditioning variable. To verify that this joint definition is necessary rather than either factor alone, we retrain the step-length model under three state definitions and compare localization on the 400 m route: action-only (four action classes), carrying-mode-only (three carrying classes), and the full joint state (twelve classes). Table 4 reports the distance-normalized error. Conditioning on either factor alone leaves substantial residual mismatch (0.71% for action-only and 0.65% for carrying-mode-only), whereas the joint state reduces the error to 0.52%. This confirms that action type and carrying mode interact, and that jointly conditioning on both is needed to capture the step-length scale rather than treating them independently.

### 4.7. Heading Fusion Ablation

To isolate the contribution of the heading module, we compare three heading sources while keeping the step detector, joint-state recognition, and step-length model (the linear variant) unchanged: gyroscope-only integration, magnetometer-only heading, and the proposed vector-domain gyroscope–magnetometer fusion. Table 5 reports the resulting trajectory metrics on the 400 m track and the circular route.

Gyroscope-only integration produces the largest error, as the integrated heading drifts without an absolute reference; magnetometer-only heading is more stable in absolute terms but remains sensitive to local magnetic disturbance. The vector-domain fusion is the most accurate on both routes, confirming that combining the short-term stability of the gyroscope with the absolute reference of the magnetometer is the dominant factor behind the heading contribution to trajectory accuracy.

### 4.8. Discussion and Limitations

The experimental results suggest that most of the improvement comes from correcting state-dependent scale mismatch in the step-length model. The fixed-parameter baseline accumulates drift when motion type or carrying mode changes, whereas the state-wise linear variant reduces this dominant error source by adapting the Weinberg coefficient to the recognized joint state. The Transformer-enhanced variant provides a smaller but consistent additional gain by smoothing coefficient changes across consecutive steps and state transitions, which is reflected in improved curvature tracking on the circular route. The remaining errors are likely associated with transition boundaries, residual heading disturbance, and user-specific gait variation.

The scope of the present evaluation is deliberately bounded. It covers planar pedestrian trajectories with controlled smartphone carrying conditions, validated on outdoor long routes and one representative indoor corridor that includes turns, building magnetic disturbance, and motion-state transitions. More demanding GPS-denied deployments, such as underground parking structures, large multi-floor buildings, and environments with persistent strong magnetic interference, are not evaluated here and are regarded as target scenarios for future study rather than validated capabilities [1,2,33]. Extending the framework to vertical-motion and multi-floor settings is a related direction. The device set is also limited to two iOS smartphones at a fixed 50 Hz sampling rate; evaluating the framework across heterogeneous hardware, including Android devices with different inertial and magnetic sensors, is left for future work.

A further limitation lies in the heading pipeline. Mapping the device-frame angular rate to the world-frame vertical axis relies on roll and pitch estimated from acceleration, which assumes that the accelerometer mainly senses gravity. Under high-dynamic motion, such as the centripetal and tangential accelerations produced during running or sharp turns, this assumption is weakened, so the tilt estimate degrades and the resulting error propagates into the heading increment and, ultimately, into the trajectory. This effect is most pronounced during vigorous motion and is one source of the residual heading error observed at transition boundaries. The heading filter also uses a fixed measurement-noise matrix R, which in regions of strong local magnetic disturbance can bias the fused heading toward the magnetometer; state- or environment-adaptive tuning of R is a natural extension. A quantitative decomposition of the total drift into step-length, heading, and state-recognition contributions is left for future work.

The available ground truth is defined at the calibration-segment and route levels rather than for individual steps, so instrumented per-step ground-truth lengths and state-resolved per-step error distributions remain directions for future evaluation. The current step detector also does not include an explicit correction mechanism for missed or false-positive step events; adding such a mechanism is left for future work.

## 5. Conclusions

This paper presents a motion-state-aware smartphone PDR method that targets frequent motion-state transitions in GPS-denied pedestrian localization. Introducing the joint motion state composed of action type and smartphone carrying mode into the PDR pipeline allows step length estimation and heading fusion to respond to different motion patterns, thereby reducing model mismatch caused by frequent motion switching.

On the step-length side, the Weinberg model is retained as the physical backbone, a state-wise linear predictor provides the lightweight adaptive coefficient model, and a compact decoder-only Transformer is evaluated as a temporal extension for per-step prediction of *K* under segment-distance supervision. On the heading side, vector-domain Kalman fusion is employed to combine gyroscope and magnetometer information, avoiding numerical instability caused by angle wrapping.

The resulting framework defines a unified comparison setting spanning fixed-parameter modeling, frequency–variance and mode-switching adaptation, linear state-wise adaptation, and a compact temporal extension for coefficient prediction. This formulation supports evaluation of how state conditioning and limited historical step-feature modeling affect coefficient estimation and long-distance trajectory consistency under frequent motion-state transitions. Future work will focus on uncertainty-aware motion state modeling, online adaptation strategies, and large-scale cross-device evaluations to further assess generalization and deployment robustness.

## Figures and Tables

**Table 1:** Summary of experimental conditions and joint motion-state categories.

Item	Description
Participants	23 participants (15 male, 8 female; height 159–189 cm), each contributing approximately 1.3 km of walking (about 30 km in total)
Devices	iPhone 14 Pro and iPhone XR (iOS); built-in tri-axial accelerometer, gyroscope, and magnetometer
Sampling rate	50 Hz (streams resampled to a unified rate for alignment)
Motion types	standing, walking, jogging, backward walking
Carrying modes	handheld, pocket, arm-swinging
Joint motion states	12 combinations (motion type × carrying mode); “standing” corresponds to a no-step state
Training routes	controlled 50 m and 100 m calibration segments covering the different joint states, used for recognition training and step-length calibration
Testing routes	400 m outdoor running track, a circular route, and an indoor corridor (≈271 m, with turns and building magnetic disturbance); the track and circular route were run once per participant (a few participants two 400 m runs), and the corridor by a subset of 4 participants; ≈3 state transitions per route
Evaluation protocol	subject-disjoint 7:3 train–test split at the participant level (16 participants for training; 7 held out for testing)
Evaluation space	planar (2D) trajectories; vertical segments excluded
Ground truth	tape-measured distances and manually annotated reference points defining a reference polyline
Metrics	point-wise distance to the reference polyline; MTE, RMSE, and distance-normalized error

**Table 2:** Classification report for joint motion-state recognition under the subject-disjoint 7:3 evaluation protocol.

Joint Motion State	Precision	Recall	F1-Score
Handheld backward walking	1.00	0.69	0.82
Handheld running	0.71	0.90	0.80
Handheld standing	0.95	0.95	0.95
Handheld walking	0.92	0.97	0.94
Pocket backward walking	0.97	0.90	0.93
Pocket running	0.73	0.93	0.82
Pocket standing	0.95	0.96	0.95
Pocket walking	0.99	0.98	0.99
Arm-swinging backward walking	0.97	0.91	0.94
Arm-swinging running	0.97	0.94	0.95
Arm-swinging standing	0.95	0.95	0.95
Arm-swinging walking	0.98	0.99	0.99
Overall accuracy	0.95		
Macro average	0.92	0.92	0.92
Weighted average	0.95	0.95	0.95

**Table 3:** Quantitative trajectory-level localization results on the three testing routes, including two adaptive step-length baselines evaluated in the same pipeline.

Route	Method	MTE (m)	RMSE (m)	Norm. Error
400 m track	Fixed baseline	5.32	6.76	1.33%
400 m track	Frequency–variance adaptive	3.80	4.26	0.95%
400 m track	Mode-switching Weinberg	3.01	3.52	0.75%
400 m track	Linear variant	2.08	2.61	0.52%
400 m track	Linear + EMA	1.93	2.45	0.48%
400 m track	Transformer variant	1.87	2.38	0.47%
Circular route	Fixed baseline	2.31	2.65	3.12%
Circular route	Frequency–variance adaptive	1.64	1.82	2.22%
Circular route	Mode-switching Weinberg	1.58	1.79	2.14%
Circular route	Linear variant	1.48	1.78	2.00%
Circular route	Linear + EMA	1.47	1.69	1.99%
Circular route	Transformer variant	1.38	1.58	1.86%
Indoor corridor	Fixed baseline	7.56	8.31	2.79%
Indoor corridor	Frequency–variance adaptive	6.99	7.97	2.58%
Indoor corridor	Mode-switching Weinberg	6.20	6.76	2.29%
Indoor corridor	Linear variant	6.01	6.63	2.22%
Indoor corridor	Linear + EMA	4.89	5.48	1.80%
Indoor corridor	Transformer variant	4.86	5.45	1.79%

**Table 4:** Effect of the state definition on 400 m localization (step-length model: linear variant).

State Definition	Norm. Error
Action only (4 classes)	0.71%
Carrying mode only (3 classes)	0.65%
Joint state (12 classes)	0.52%

**Table 5:** Heading-fusion ablation on the 400 m track and the circular route, with the step-length model fixed to the linear variant.

Heading Source	Route	MTE (m)	RMSE (m)	Norm. Error
Gyroscope only	400 m track	5.12	5.84	1.28%
Magnetometer only	400 m track	3.45	4.12	0.86%
Vector-domain fusion	400 m track	2.08	2.61	0.52%
Gyroscope only	Circular route	4.85	5.60	6.55%
Magnetometer only	Circular route	3.22	3.88	4.35%
Vector-domain fusion	Circular route	1.48	1.78	2.00%

## Data Availability

The datasets and code supporting the findings of this study are available from the corresponding author upon reasonable request. The materials are not publicly available at this stage because the dataset is still being organized and curated for release.
